# Maternal Bleeding Outcomes in Pregnant Women With Immune Thrombocytopenia (ITP): A National Database Study

**DOI:** 10.7759/cureus.110602

**Published:** 2026-06-10

**Authors:** Marian Araji, Ibrahim Al Saidi, Zaid Khamis, Ahmad Mustafa, Zahraa Ajami, Dima Ezzedine, Ghada Araji, Alexander Bershadskiy

**Affiliations:** 1 Department of Medicine, Poznan University of Medical Sciences, Poznan, POL; 2 Department of Internal Medicine, Northwell Health/Staten Island University Hospital, New York, USA; 3 Department of Cardiology, Northwell Health/Staten Island University Hospital, New York, USA; 4 Department of Medicine, University of Georgia, School of Health Sciences, Tbilisi, GEO; 5 Department of Medicine, Gilbert and Rose-Marie Chagoury School of Medicine, Lebanese American University, Byblos, LBN; 6 Department of Hematology and Oncology, Northwell Health/Staten Island University Hospital, New York, USA

**Keywords:** immune thrombocytopenic purpura, maternal outcomes, mortality, postpartum hemorrhage, pregnancy

## Abstract

Objective: Immune thrombocytopenic purpura (ITP) is a hematological disorder commonly affecting women of reproductive age, but limited studies explore the maternal morbidity in pregnant patients with ITP. This study aimed to investigate whether pregnant women with ITP are at higher risk of bleeding complications during and after delivery.

Methods: We analyzed the National Inpatient Sample (NIS) database for pregnant patients admitted for delivery from 2016 to 2018. Patients were stratified into two cohorts: those with ITP and those without. The two cohorts were matched in a 1:1 ratio based on multiple factors. Univariate and binary logistic regression analyses were used to evaluate outcomes, including intrapartum hemorrhage, postpartum hemorrhage, transfusion requirements, and mode of delivery.

Results: Among 207,511 pregnant patients, 215 had ITP. Intrapartum hemorrhage was more frequent in the ITP cohort, though not statistically significant (0.9% vs. 0.3%; p=0.107). In contrast, postpartum hemorrhage was significantly higher in patients with ITP (10.2% vs. 4.4%; p<0.001). Transfusion requirements were similar between the two groups (2.3% vs. 1.4%; p=0.223). Mode of delivery did not differ significantly between cohorts (p=0.883), though patients with ITP requiring platelet transfusions were more likely to undergo C-section (83.3% vs. 51.5%; p=0.03). Intravenous immunoglobulin use did not significantly impact delivery mode (p=0.516).

Conclusion: Pregnant patients with ITP have a significantly higher risk of postpartum hemorrhage, emphasizing the need for vigilant monitoring and proactive management. These findings highlight the importance of heightened peripartum surveillance and individualized management strategies to mitigate bleeding risk in this population.

## Introduction

Immune thrombocytopenia (ITP) is an autoimmune disorder characterized by isolated thrombocytopenia, defined as a peripheral blood platelet count below 100 x 10^9^/L, in the absence of other identifiable causes of thrombocytopenia [1]. It affects individuals across all age groups but is more prevalent in women of childbearing age. When ITP occurs in pregnancy, it poses unique challenges for both maternal and fetal health, as the disease’s course can be unpredictable during this period [2]. Some pregnant women with a history of ITP will experience worsening of their condition during gestation, while others may observe an improvement [3]. The incidence of ITP in pregnancy is estimated at 1 to 2 per 1,000 pregnancies, accounting for approximately 3% of all cases of thrombocytopenia in pregnancy, which makes it a relatively rare but significant concern in obstetric care [4]. Clinical presentation can vary widely, ranging from asymptomatic thrombocytopenia to severe bleeding complications, with bleeding risk only partially correlated to the degree of thrombocytopenia [5,6]. While numerous studies have examined neonatal outcomes in pregnancies complicated by ITP, limited data exist on maternal comorbidity, particularly intrapartum and postpartum hemorrhage [7,8]. Current management guidelines for ITP in pregnancy are largely based on expert opinion and retrospective studies, leaving significant gaps in evidence-based practice. As a result, the magnitude of maternal bleeding risk associated with ITP during pregnancy remains incompletely defined. Using a large national inpatient database, the present study seeks to address this knowledge gap by evaluating the association between ITP and intrapartum hemorrhage, postpartum hemorrhage, and delivery characteristics in pregnant patients. By leveraging a large sample size and real-world national data, this study provides a broader assessment of maternal outcomes and may help inform obstetric and hematologic management strategies for pregnant patients with ITP.

## Materials and methods

We conducted a retrospective cohort study using the National Inpatient Sample (NIS) database from 2016 to 2018. The NIS, part of the Healthcare Cost and Utilization Project (HCUP), is the largest publicly available inpatient database in the United States. Because the dataset is fully de-identified and HIPAA-compliant, institutional review board approval and informed consent were not required.

Formal sample size estimation was not performed because this study used an administrative database and included all eligible delivery admissions during the pre-specified study interval, representing exhaustive consecutive sampling within the database. Pregnant patients admitted for delivery between 2016 and 2018 were identified using delivery-related ICD-10 diagnosis and procedure codes from the original study query. Patients were then stratified into two cohorts based on the presence or absence of ITP. Patients without delivery-related admissions and records missing key analytic variables were not eligible for analysis. Additionally, patients with other identifiable causes of thrombocytopenia, when distinguishable using ICD-10 codes (e.g., thrombotic thrombocytopenic purpura, HELLP syndrome, or disseminated intravascular coagulation), were excluded.

ICD-10 codes were used to identify ITP, intrapartum hemorrhage, postpartum hemorrhage, transfusion, mode of delivery, and in-hospital mortality. The primary outcomes were intrapartum hemorrhage and postpartum hemorrhage. Secondary outcomes included blood transfusion requirements, mode of delivery, and mortality. To reduce confounding, the two cohorts were matched based on key baseline characteristics, including age, race, antiplatelet use, anticoagulant use, and steroid use. 

Statistical analysis 

**Figure 1:**
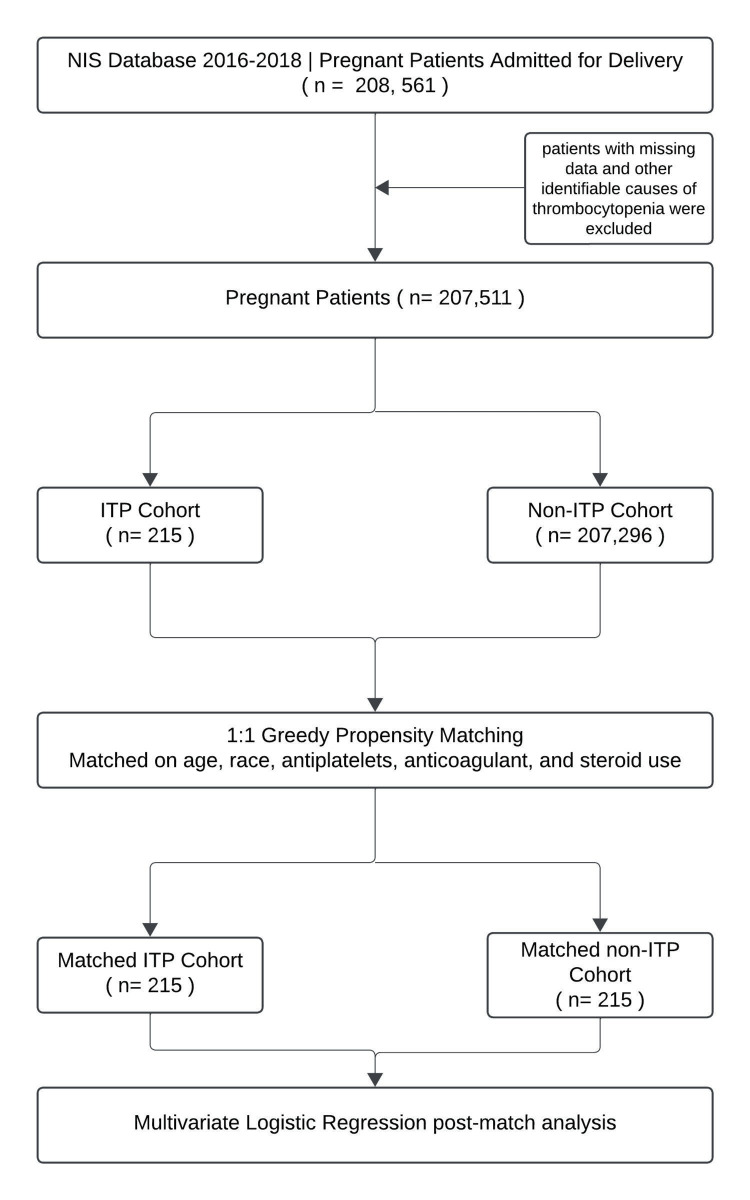
Study flow diagram Patients were identified using ICD-10 codes and stratified into ITP and non-ITP cohorts, followed by 1:1 propensity score matching. The diagram has been created using Lucidchart. ITP, immune thrombocytopenic purpura; NIS, National Inpatient Sample.

The two groups were initially compared to assess the outcomes. Continuous variables were analyzed using Student's t-tests. Categorical variables were analyzed using chi-square analyses and Fisher's exact tests. The two groups were then matched 1:1 to account for potential confounding variables. Greedy propensity matching was performed using R version 4.1.2 (R Foundation for Statistical Computing, Vienna, Austria; 2021). Pregnant patients with ITP were matched to those without ITP for age, race, antiplatelet use, anticoagulant use, and steroid use. Post-match p-values were >0.05, signifying successful matching. Binary logistic regression was then performed on the matched cohorts to estimate ORs and 95% CIs. All statistical analyses, besides propensity score matching, were performed using IBM SPSS Statistics for Windows, version 28 (IBM Corp., Armonk, NY, USA; 2021). p-values <0.05 were considered statistically significant. The study design is illustrated in Figure 1.

## Results

A total of 207,511 pregnant patients admitted for delivery were identified, including 215 (0.1%) patients with ITP. Baseline characteristics of the study population are summarized in Table 1.

**Table 1:** Baseline characteristics of pregnant patients admitted for delivery (total N=207,511) Data are presented as numbers (%) for categorical variables and means for continuous variables. Categorical variables were analyzed using chi-square or Fisher’s exact test, and continuous variables were analyzed using Student’s t-test. Reported p-values correspond to these statistical tests. ITP, immune thrombocytopenia.

Characteristic	ITP (n=215)	Non-ITP (n=207,296)	p-value
Mean age	30.27	29.49	0.046
Race			0.526
White	102 (47.4%)	96,821 (46.7%)	
Black	53 (24.7%)	48,752 (23.5%)	
Hispanic	47 (21.9%)	46,070 (22.2%)	
Asian or Pacific	2 (0.9%)	5,971 (2.9%)	
Native American	1 (0.5%)	2,069 (1%)	
Other	10 (4.7%)	7,613 (3.7%)	
Oral anticoagulant use	8 (3.7%)	1,206 (0.6%)	<0.001
Aspirin use	7 (3.3%)	4,737 (2.3%)	0.341
Antiplatelet use	0 (0%)	104 (0.1%)	0.743
Steroid use	6 (2.8%)	218 (0.1%)	<0.001

In the unmatched analysis, intrapartum hemorrhage was more frequent in the ITP cohort than in the non-ITP cohort, although this difference did not reach statistical significance (0.9% vs. 0.3%; p=0.107). In contrast, postpartum hemorrhage was significantly higher in patients with ITP (10.2% vs. 4.4%; p<0.001). Blood transfusion requirements were not significantly different between groups (2.3% vs. 1.4%; p=0.223) (Table 2).

**Table 2:** Outcomes in unmatched cohorts Data are presented as numbers (%). Categorical variables were analyzed using chi-square or Fisher’s exact test, as appropriate. Reported p-values correspond to these statistical tests. ITP, immune thrombocytopenia.

Outcome	ITP (n=215)	Non-ITP (n=207,296)	p-value
Postpartum hemorrhage	22 (10.2%)	9,146 (4.4%)	<0.001
Intrapartum hemorrhage	2 (0.9%)	651 (0.3%)	0.107
Blood transfusion requirements	5 (2.3%)	2,824 (1.4%)	0.223

**Figure 2:**
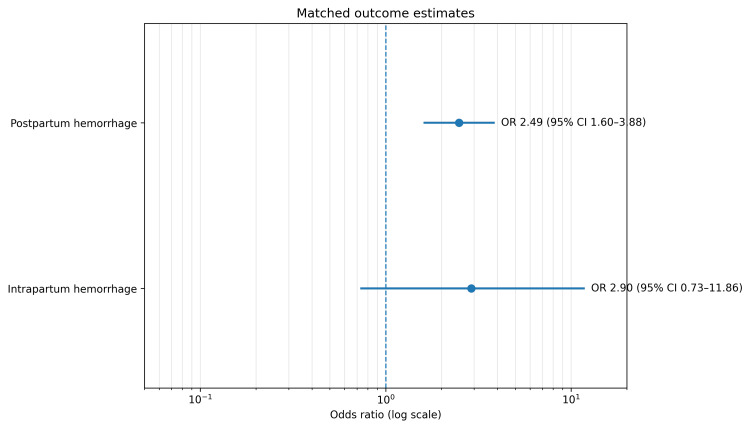
Forest plot of ORs for intrapartum and postpartum hemorrhage in pregnant patients with ITP following propensity score matching ORs and 95% CIs are shown for intrapartum hemorrhage and postpartum hemorrhage following 1:1 propensity score matching. The vertical dashed line at OR=1 represents no difference between groups. The figure has been created using Microsoft Excel (Microsoft Corporation, Redmond, WA, USA).

After 1:1 propensity score matching, 215 patients were included in each group. The magnitude and direction of associations remained consistent with the unmatched analysis. Intrapartum hemorrhage remained higher in the ITP cohort but did not reach statistical significance (OR: 2.9; 95% CI: 0.72-11.86; p=0.131). Postpartum hemorrhage remained significantly increased in patients with ITP (OR: 2.49; 95% CI: 1.6-3.88; p<0.001) (Table 3). These associations are illustrated in Figure 2. No mortality events were observed in the ITP cohort, while 29 deaths occurred in the non-ITP group. Due to the absence of events in the ITP group, comparative analysis for mortality was not performed.

**Table 3:** Outcomes after propensity score matching (n=215 per group) Data are presented as ORs with 95% CIs. Logistic regression analysis was used to estimate ORs and corresponding p-values.

Outcome	OR	95% CI	p-value
Postpartum hemorrhage	2.49	1.6-3.88	<0.001
Intrapartum hemorrhage	2.9	0.72-11.86	0.131

The presence of ITP was not associated with differences in mode of delivery. Rates of cesarean and vaginal delivery were similar between ITP and non-ITP cohorts (p=0.883) (Table 4). Among patients with ITP, 12 (5.6%) received platelet transfusions and 6 (2.8%) received intravenous immunoglobulin (IVIG) before delivery (Table 5). Patients who received platelet transfusions had a higher rate of cesarean delivery than those who did not (83.3% vs. 51.5%; p=0.03), whereas IVIG use was not associated with delivery mode (p=0.516) (Tables 6, 7). These subgroup analyses should be interpreted with caution due to small sample sizes.

**Table 4:** Mode of delivery in patients with and without ITP Data are presented as numbers (%). Categorical variables were analyzed using chi-square or Fisher’s exact test, as appropriate. Reported p-values correspond to these statistical tests. ITP, immune thrombocytopenia.

Delivery mode	ITP (n=177)	Non-ITP (n=189,934)	p-value
Vaginal delivery	82 (46.3%)	89,544 (47.1%)	0.883
Cesarean section	95 (53.6%)	100,390 (52.9%)	

**Table 5:** Pre-delivery treatment in patients with ITP (n=215) Data are presented as numbers (%). IVIG, intravenous immunoglobulin.

Treatment	Number (%)
Platelet transfusion	12 (5.6%)
IVIG	6 (2.8%)
Both	1 (0.5%)

**Table 6:** Association between platelet transfusion and mode of delivery in patients with ITP Data are presented as numbers (%). Categorical variables were analyzed using chi-square or Fisher’s exact test, as appropriate. Reported p-values correspond to these statistical tests.

Delivery mode	Platelet transfusion (n=12)	No transfusion (n=165)	p-value
Vaginal delivery	2 (16.7%)	80 (48.5%)	0.03
Cesarean section	10 (83.3%)	85 (51.5%)	

**Table 7:** Association between IVIG use and mode of delivery in patients with ITP Data are presented as numbers (%). Categorical variables were analyzed using chi-square or Fisher’s exact test, as appropriate. Reported p-values correspond to these statistical tests. IVIG, intravenous immunoglobulin.

Delivery mode	IVIG (n=6)	No IVIG (n=171)	p-value
Vaginal delivery	2 (33.3%)	80 (46.8%)	0.516
Cesarean section	4 (66.7%)	91 (53.3%)	

## Discussion

In this large national cohort study, pregnant patients with ITP had a significantly higher risk of postpartum hemorrhage than those without ITP. This association remained consistent after propensity score matching, with an approximately 2.5-fold increase in risk. In contrast, intrapartum hemorrhage was not significantly different between groups, although a nearly threefold numerical increase was observed. The wide CI and the rarity of intrapartum hemorrhage events in our cohort suggest that this analysis was underpowered to detect a true difference, and the absence of statistical significance should not be interpreted as evidence against an association between ITP and intrapartum bleeding risk.

Several studies have evaluated pregnancy outcomes in patients with ITP; however, few have specifically focused on maternal bleeding complications during delivery [9-11]. Prior prospective data have demonstrated that pregnancy is associated with increased ITP exacerbations and treatment requirements, although overall bleeding rates remain relatively low and severe bleeding is uncommon (HR: 1.83; 95% CI: 0.91-3.65). Importantly, this study compared pregnant patients with ITP to non-pregnant patients with ITP, rather than to pregnant patients without ITP, limiting their ability to assess delivery-related bleeding risk [9]. In contrast, our study provides a direct comparison between pregnant patients with and without ITP at a national level and demonstrates a significantly increased risk of postpartum hemorrhage (10.2% vs. 4.4%; OR: 2.49). The incidence of postpartum hemorrhage observed in our ITP cohort (10.2%) is consistent with prior reports, which have documented rates ranging from approximately 9% to over 50% in patients with ITP [12-15]. Similarly, a recent meta-analysis reported a postpartum hemorrhage rate of 11% and a cesarean section rate of 48% among pregnant patients with ITP, both of which closely align with our findings [16]. 

Despite the increased risk of postpartum hemorrhage, we did not observe significant differences in transfusion requirements between groups (2.3% vs. 1.4%; p=0.223). While this may suggest that bleeding events in patients with ITP do not uniformly result in transfusion-requiring hemorrhage, this finding should be interpreted with caution. The NIS database lacks detailed clinical indicators of hemorrhage severity, such as estimated blood loss, hemoglobin changes, use of uterotonic agents, or need for procedural interventions, and therefore transfusion utilization alone may not fully capture the clinical burden of postpartum hemorrhage in this population. Similar findings have been reported in several prior studies [10,13,17,18]. In the UK national cohort study by Care et al., women with severe primary autoimmune thrombocytopenia had a 21% incidence of postpartum hemorrhage, yet the majority of bleeding episodes were managed conservatively without requiring significant transfusion support [13]. Similarly, Ezveci et al. reported that despite higher rates of bleeding complications in pregnant patients with ITP at a single tertiary center, transfusion requirements did not differ significantly from controls [17]. One possible explanation for this dissociation is that postpartum hemorrhage in ITP may often be mild to moderate in severity and responsive to conservative management strategies. A 2023 review by Bussel and colleagues highlighted that severe bleeding in pregnant patients with ITP is uncommon and that postpartum hemorrhage does not appear to be increased with appropriate management, emphasizing the importance of optimizing platelet counts in the peripartum period [4]. Furthermore, the ACOG Practice Bulletin No. 207 emphasizes that platelet transfusions in ITP should be reserved as a temporary measure for life-threatening hemorrhage or preparation for urgent surgery, as the effect on platelet count is short-lived due to accelerated platelet destruction [19]. This conservative approach to transfusion in ITP may partly explain the similar transfusion rates observed despite higher hemorrhage incidence. However, these results should be interpreted cautiously, as transfusion practices may vary across institutions and are influenced by clinical judgment, local protocols, and patient-specific factors. 

Our study demonstrated that the presence of ITP was not associated with differences in mode of delivery, as rates of cesarean and vaginal delivery were comparable between ITP and non-ITP cohorts. This finding suggests that ITP alone is not an independent determinant of delivery method and that obstetric indications remain the primary drivers of delivery decisions [19]. However, among patients with ITP, those who received platelet transfusions had significantly higher rates of cesarean delivery. This association likely reflects confounding by indication, as platelet transfusions are typically administered in patients with more severe thrombocytopenia or in anticipation of invasive procedures. In clinical practice, platelet thresholds often guide delivery planning, with a target platelet count of at least 50 x 10^9^/L commonly recommended for cesarean delivery to minimize surgical bleeding risk [20]. As a result, patients with lower platelet counts may be more likely to receive transfusions and subsequently undergo cesarean delivery as indicated. Additionally, clinicians may preferentially select cesarean delivery in patients with significant thrombocytopenia to allow for a more controlled environment, where bleeding risks can be anticipated and managed more effectively. Therefore, the observed association between platelet transfusion and cesarean delivery likely reflects both underlying disease severity and procedural planning rather than a direct causal relationship.

In contrast, IVIG use was not associated with differences in delivery mode. This may be explained by the fact that IVIG is often administered in a planned, non-urgent setting to gradually increase platelet counts before delivery, allowing for optimization of platelet counts irrespective of the anticipated mode of delivery. Unlike platelet transfusions, which are frequently used in acute or peri-procedural contexts, IVIG may reflect more stable disease management and therefore may not directly influence delivery decisions. These subgroup findings should be interpreted with caution given the small sample sizes.

The relationship between platelet count and bleeding risk in pregnancy remains complex. While thrombocytopenia is a known risk factor for postpartum hemorrhage, bleeding risk is influenced by multiple obstetric factors, including uterine atony, placental abnormalities, and labor-related variables [7,21]. Notably, prior studies have demonstrated that platelet count is not always directly correlated with bleeding severity in pregnant patients with ITP, highlighting the multifactorial nature of hemorrhagic risk [22-24]. In our study, we were unable to assess platelet counts or disease severity, which are important determinants of bleeding risk. Future studies incorporating laboratory data and disease-specific variables would help better characterize these relationships.

This study has several important strengths. The use of a large, nationally representative database enhances the generalizability of our findings and allows for evaluation of relatively rare conditions such as ITP in pregnancy. Additionally, the use of propensity score matching helps reduce confounding and enables a more balanced comparison between patients with and without ITP. Importantly, this study directly compares maternal bleeding outcomes between these groups at a national level, addressing a gap in the existing literature, as prior studies have largely focused on neonatal outcomes or lacked appropriate control groups. However, several limitations should be considered. The use of administrative ICD-10 codes introduces the potential for misclassification bias, and important clinical variables such as platelet count, severity of ITP, timing and indication for treatment, and detailed obstetric factors were not available. Additionally, residual confounding from unmeasured variables may influence bleeding risk. The analysis of mortality and subgroup outcomes was limited by low event numbers, which may reduce the precision of these estimates. Despite these limitations, this study contributes meaningful evidence to an area with limited data, particularly regarding maternal bleeding risk during delivery in patients with ITP. 

## Conclusions

In this large national cohort study, pregnant patients with ITP had a significantly higher risk of postpartum hemorrhage than those without ITP, while transfusion requirements, mode of delivery, and mortality remained comparable between groups. These findings suggest that although ITP increases the frequency of postpartum bleeding events, current peripartum management strategies appear effective in preventing escalation to severe maternal outcomes. These results reinforce the importance of multidisciplinary peripartum planning and heightened postpartum surveillance in pregnant patients with ITP. Future prospective studies incorporating platelet counts, disease severity, treatment parameters, and standardized bleeding assessment tools will be essential to further refine risk stratification and optimize management in this population.

## Appendices

**Table 8:** Supplementary table of ICD-10 diagnosis codes used for cohort identification and outcome definitions This table summarizes the ICD-10 diagnosis codes used for cohort identification and outcome ascertainment. Immune thrombocytopenia (ITP) was identified using ICD-10 code D69.3. Maternal hemorrhagic outcomes included intrapartum hemorrhage (O67.8, O67.9) and postpartum hemorrhage (O72.0, O72.1, O72.2). These codes were applied uniformly across the study cohort to identify exposures and outcomes of interest.

Variable	ICD-10 code	Description
Immune thrombocytopenia (ITP)	D69.3	Immune thrombocytopenic purpura
Intrapartum hemorrhage	O67.8	Other intrapartum hemorrhage
Intrapartum hemorrhage	O67.9	Intrapartum hemorrhage, unspecified
Postpartum hemorrhage	O72.0	Third-stage hemorrhage
Postpartum hemorrhage	O72.1	Other immediate postpartum hemorrhage
Postpartum hemorrhage	O72.2	Delayed and secondary postpartum hemorrhage
